# Long-Term Swallowing Outcomes Following Thyroidectomy: Clinical Determinants of Persistent Swallowing Symptoms in Endocrine Surgical Oncology Care

**DOI:** 10.3390/healthcare14162639

**Published:** 2026-08-20

**Authors:** Yavuz Selim Kahraman, Fatma Gün, Nursu Başakcioğlu, Ahmet Buğra Örtlek, Yasin Alper Yıldız, Adem Şentürk

**Affiliations:** 1 Department of General Surgery, Division of Surgical Oncology, Kastamonu Training and Research Hospital, Kastamonu 37150, Türkiye; 2 Department of General Surgery, Kastamonu University, Kastamonu 37150, Türkiye; cetinfatmagun@gmail.com (F.G.); nursusahar@hotmail.com (N.B.); yalperyildiz@gmail.com (Y.A.Y.); 3 Department of General Surgery, Kastamonu Training and Research Hospital, Kastamonu 37150, Türkiye; bugraortlek@gmail.com; 4 Department of Surgical Oncology, Sakarya Training and Research Hospital, Sakarya 54100, Türkiye; dr.adem.senturk@gmail.com

**Keywords:** endocrine surgical procedures, surgical oncology, dysphagia, thyroidectomy, deglutition

## Abstract

**Highlights:**

**What are the main findings?**

- Patients discharged after thyroidectomy surgery experienced significant improvement in swallowing function during the first year following the procedure.
- Determinants for persistent patient-reported swallowing symptoms include preoperative and postoperative dysphonia, high body mass index, and malignant pathology. The rate of patients developing persistent patient-reported swallowing symptoms was 8.6%.

**What are the implications of the main findings?**

- Patients experiencing dysphonia before or after surgery should be monitored more closely for early diagnosis of persistent swallowing symptoms.
- Whether or not they have cancer, recognizing clinical features and risk factors, informing the patient, and providing individualized care are crucial for patients undergoing thyroid surgery.

**Abstract:**

Background and Objectives: This study was conducted to assess changes in swallowing function in thyroidectomy patients and to identify clinical risk factors associated with persistent patient-reported swallowing symptoms. Methods: The study was a retrospective, observational study. Data were collected from 280 patients who underwent total thyroidectomy between January 2022 and January 2025. Swallowing function was assessed using the Turkish Eating Assessment Tool-10 (T-EAT-10). Multivariate logistic regression analysis was employed to identify independently associated factors for persistent patient-reported swallowing symptoms, and receiver operating characteristic (ROC) analysis was used to assess the discriminative performance of continuous variables. Results: Patient-reported T-EAT-10 scores changed significantly during the follow-up period (χ^2^ = 217.33, *p* < 0.001). Post hoc analyses revealed that dysphagia scores decreased significantly at the 6th and 12th months postoperatively (*p* < 0.001). Persistent patient-reported swallowing symptoms were detected in 24 (8.6%) out of 279 patients who had a full 12-month T-EAT-10 follow-up. Multivariate logistic regression analysis showed that postoperative and preoperative dysphonia, body mass index (BMI), and malignant pathology were independently associated with persistent patient-reported swallowing symptoms. In the ROC analysis, the approximate clinical exploratory threshold values were 29 mm (AUC = 0.692) for nodule size and 100 min (AUC = 0.660) for operation time. Internal cross-validation analysis showed that the model’s discriminative performance was preserved, with a mean cross-validated AUC value of 0.801. Conclusions: Dysphagia following thyroidectomy typically resolves over time. However, the presence of preoperative and postoperative dysphonia appears to be a strong indicator of the development of persistent patient-reported swallowing symptoms.

## 1. Introduction

Thyroidectomy is a safe surgical procedure that is widely used to treat benign and malignant thyroid diseases. However, functional symptoms that develop during the postoperative period can significantly impact patients’ quality of life. Notable postoperative symptoms, including voice changes, a sensation of something being stuck in the throat (globus sensation), and difficulty swallowing, can occur even in the absence of serious complications, such as recurrent laryngeal nerve injury [1,2].

Although dysphagia is more commonly linked to laryngeal nerve injury, recent studies suggest that swallowing disorders can arise even following uncomplicated surgeries. This suggests that postoperative dysphagia cannot be explained solely by nerve damage but that multiple mechanisms, such as surgical dissection, strap muscle manipulation, laryngotracheal mobilization, edema, fibrosis, and changes in upper esophageal sphincter function, may also play a role [3,4].

The literature reports that dysphagia following thyroidectomy is common and often improves over time for many patients [5]. However, for some patients, symptoms may persist for months, having a lasting impact on their stated quality of life [6,7]. For this reason, post-thyroidectomy dysphagia should be considered not only an early surgical complication but also in terms of long-term functional outcomes.

Patient-reported outcomes are increasingly important in assessing postoperative dysphagia. Measurement tools have been developed for this purpose. For example, the Eating Assessment Tool-10 (EAT-10) is a brief, validated, and reliable measure of dysphagia symptoms [8]. The Turkish version, the T-EAT-10, has also been reported to be valid and reliable for assessing dysphagia in adult patients [9]. Furthermore, another study demonstrated that a threshold value of ≥3 for the EAT-10 provides greater accuracy in dysphagia screening [10].

In this context, particularly in cases of malignant thyroid disease, the need for broader surgical dissection and more intensive manipulation of surrounding anatomical structures is an important factor in terms of functional outcomes. Therefore, assessing swallowing function after thyroidectomy is a key parameter that reflects postoperative quality of life [11,12]. The American Thyroid Association (ATA) guidelines play a significant role in determining the extent of surgery, particularly in cases of differentiated thyroid cancer. The evaluation of functional outcomes is also important for patients requiring broader surgical dissection [13].

However, studies in the current literature assessing dysphagia following thyroidectomy and identifying independent clinical risk factors associated with the development of persistent patient-reported swallowing symptoms during long-term follow-up are limited. Of particular interest are the long-term effects on swallowing function of self-reported preoperative and postoperative dysphonia, difficult dissection, malignant pathology, and variables related to the surgical process. The objective of this study was therefore to evaluate temporal changes in swallowing function using the T-EAT-10 score in patients who underwent thyroidectomy and to identify clinical risk factors associated with persistent patient-reported swallowing symptoms at the 12-month follow-up.

## 2. Materials and Methods

### 2.1. Study Design

This retrospective observational study examined patients who underwent thyroidectomy at our clinic between January 2022 and January 2025. Ethical committee approval and institutional authorization for this study were obtained from the Kastamonu University Faculty of Medicine Non-Interventional Clinical Research Ethics Committee (date: 18 December 2025, approval number: 2025-40). Data were collected between January and May 2026. The study was performed in accordance with the principles of the Declaration of Helsinki (1964).

### 2.2. Study Population and Setting

The study enrolled 280 patients who underwent total thyroidectomy between January 2022 and January 2025. Experienced endocrine surgeons performed all surgical procedures. During the operation, care was taken to preserve the recurrent laryngeal nerve and the parathyroid glands. Complications such as postoperative dysphonia, hypocalcemia, and cervical hematoma were recorded.

Postoperative dysphonia was assessed based on any change in voice following surgery, including vocal fatigue, reduced vocal strength, breathy voice, or difficulty projecting the voice. Patients with preoperative vocal cord paralysis were not included in the study. However, although standardized preoperative and postoperative laryngeal examinations were performed on all patients, structured voice assessments were not systematically determined for all patients. Therefore, dysphonia was defined according to patient-reported voice changes during routine clinical follow-up. Hypocalcemia was defined as a postoperative serum calcium level falling below the laboratory reference range and/or the need for calcium replacement therapy. Nodule sizes were assessed based on ultrasound and pathology reports. Nodule sizes were divided into three groups: <20 mm (Group 0), 20–40 mm (Group 1), and >40 mm (Group 2).

### 2.3. Inclusion Criteria and Exclusion Criteria

The inclusion criteria included being aged 18 years or over, having undergone total thyroidectomy due to benign or malignant thyroid disease, and having at least 12 months of regular postoperative follow-up data. The exclusion criteria included having previously undergone neck surgery, preoperative vocal cord paralysis, dysphagia due to another condition, or a history of esophageal motility disorder.

### 2.4. Data Collection

Demographic and clinical data were retrieved from the electronic patient record system. The data were evaluated retrospectively, and the patients to be included in the study were identified in advance. The following data were collected retrospectively: age; sex; body mass index (BMI); smoking status; presence of comorbidities; thyroid-stimulating hormone (TSH) level; nodule size; weight of thyroidectomy specimen; duration of surgery; length of hospital stay; and contact information. Subsequently, the patients’ pathological findings were classified as benign or malignant. The malignant cases were then subdivided into the following types of thyroid carcinoma: papillary (PTC), papillary micro (PTMC), follicular (FTC), medullary (MTC), and non-invasive follicular thyroid neoplasia with papillary-like nuclear features (NIFTP).

For assessment of swallowing function, the validated and reliable Turkish version of the T-EAT-10 [9] was used to assess postoperative swallowing function. Following institutional and ethics approval, the T-EAT-10 questionnaire was administered retrospectively, and patients were asked to recall their dysphagia function before surgery and at 1, 6, and 12 months after thyroidectomy. Some patients completed the questionnaire during routine outpatient follow-up, whereas others completed it by telephone. The scale comprises 10 items, each scored from 0 to 4, resulting in a total score ranging from 0 to 40. Higher scores indicate more severe symptoms of dysphagia. In accordance with the threshold value recommended in the literature, a T-EAT-10 score of ≥3 was considered indicative of dysphagia [10]. In this study, persistent patient-reported swallowing symptoms were indicated by a T-EAT-10 score. A score of ≥3 at the 12-month postoperative follow-up was defined as “persistent dysphagia”. This definition and exploratory threshold value reflect symptoms reported by patients rather than an objectively confirmed physiological impairment.

### 2.5. Statistical Analyses

Statistical analyses were performed using IBM SPSS Statistics (version 26.0). Continuous variables are expressed as the mean ± standard deviation or the median. Categorical variables are expressed as counts and percentages. The Kolmogorov–Smirnov test was used to assess the normality of continuous variables. Two independent groups were compared using a Student’s *t*-test or a Mann–Whitney U test, depending on normality. The chi-square test or, when appropriate, Fisher’s exact test was used to compare categorical variables. When significant differences were identified, the Wilcoxon signed-rank test was used for pairwise comparisons, and the Bonferroni correction was applied for multiple comparisons. A multivariate logistic regression analysis was performed to identify factors associated with persistent patient-reported swallowing symptoms. Variables with *p* < 0.10 in the univariate analysis that were also considered clinically significant were included in the multivariate model using the enter method. Results are reported as odds ratios (ORs) and 95% confidence intervals (CIs). Receiver operating characteristic (ROC) curve analysis was performed to evaluate the discriminatory performance of continuous variables in predicting persistent patient-reported swallowing symptoms. The area under the curve (AUC), 95% confidence intervals, sensitivity, specificity, and optimal exploratory threshold values were calculated using the Youden index. In all analyses, a *p*-value of <0.05 was deemed statistically significant.

## 3. Results

### 3.1. Patient Characteristics

A total of 280 patients were included in the study. The mean age of the patients was 52.68 ± 11.13 years, and the median age was 53 years, with an observed range of 27–78 years. Body mass index (BMI) was 25.99 ± 5.47 kg/m^2^. Of these patients, 219 (78.2%) were female, and 61 (21.8%) were male. The smoking prevalence was found to be 25%. At least one comorbidity was present in 52.1% of patients.

Pathological examination found that 72.1% of cases were benign, while 27.9% were malignant. Among the malignant cases, papillary thyroid microcarcinoma (14.3%) and papillary thyroid carcinoma (9.3%) were the most prevalent. When evaluated by nodule size, more than half of the patients (51.4%) were in the 20–40 mm group (see Table 1).

### 3.2. Temporal Changes in T-EAT-10 Scores

Mean score evaluation revealed that dysphagia symptoms persisted markedly in the early postoperative period but progressively decreased during the follow-up period. The Friedman analysis revealed a significant change in T-EAT-10 scores over the follow-up period (χ^2^ = 217.33, *p* < 0.001) (see Table 2). Bonferroni-adjusted pairwise Wilcoxon signed-rank tests demonstrated a negligible effect between the preoperative assessment and postoperative month 1 (r = 0.03), moderate effects for comparisons involving postoperative months 6 and 12 (r = 0.38–0.42), and large effects for comparisons between postoperative month 1 and months 6 and 12 (r = 0.58–0.62), indicating clinically meaningful longitudinal improvement in patient-reported swallowing symptoms.

### 3.3. Factors Related to Persistent Patient-Reported Swallowing Symptoms

At the 12-month postoperative follow-up, persistent patient-reported swallowing symptoms were observed in 24 patients (8.6%). Patients who developed persistent patient-reported swallowing symptoms had a significantly longer hospital stay (1.83 ± 0.82 days vs. 1.28 ± 0.50 days, *p* < 0.001). Additionally, operative time and nodule size were significantly higher in these patients (*p* = 0.005 and *p* = 0.002, respectively). BMI values were higher in patients who developed dysphagia, and this difference showed borderline statistical significance in the univariate analysis (*p* = 0.050). No significant differences were observed between the groups in terms of thyroid weight, age, or TSH levels (see Table 3).

### 3.4. Multivariate Logistic Regression Analysis

The multivariate logistic regression model was significant overall (χ^2^ = 43.06, *p* < 0.001). Based on the Hosmer–Lemeshow test (χ^2^ = 11.19, df = 8, *p* = 0.191), model calibration was found to be acceptable, and the Nagelkerke R^2^ value was calculated as 0.322. The analysis revealed that postoperative and preoperative dysphonia, BMI, and malignant pathology were independently associated with persistent patient-reported swallowing symptoms.

**Figure 1:**
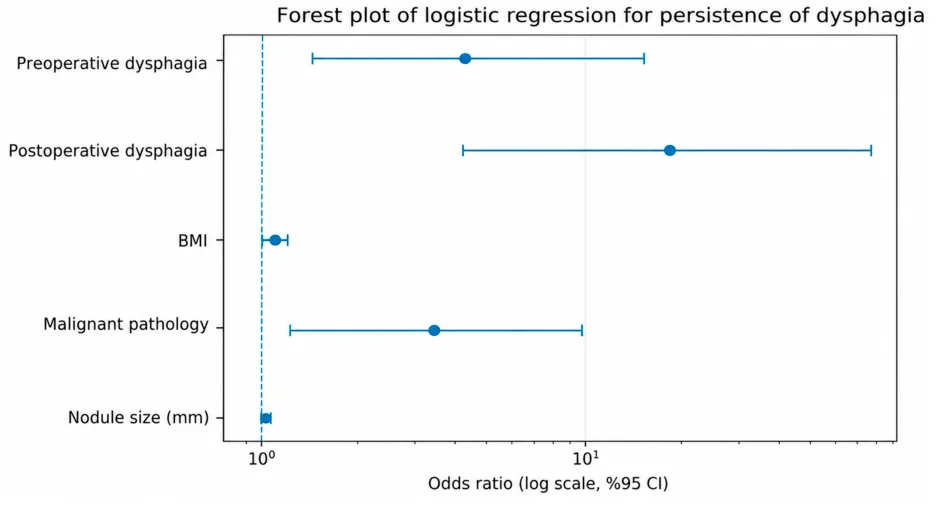
Multivariate logistic regression forest plot for persistent patient-reported swallowing symptoms.

Postoperative dysphonia was found to be the strongest independently associated factor for persistent patient-reported swallowing symptoms (OR = 17.54, 95% CI: 4.13–74.56, *p* < 0.001). Additionally, preoperative dysphonia was found to significantly increase the risk of dysphagia (OR = 4.40, 95% CI: 1.42–13.66, *p* = 0.010). Although a high BMI was found to be an independently associated factor for dysphagia, the effect size was relatively small. While BMI showed only borderline significance in the univariate analysis, the fact that the association became more pronounced in the multivariate analysis suggests that the effect of this variable may be influenced by interactions with other clinical and surgical factors. The presence of malignant pathology was also found to be an independently associated factor for persistent patient-reported swallowing symptoms (see Table 4, Figure 1).

### 3.5. ROC Analysis

**Figure 2:**
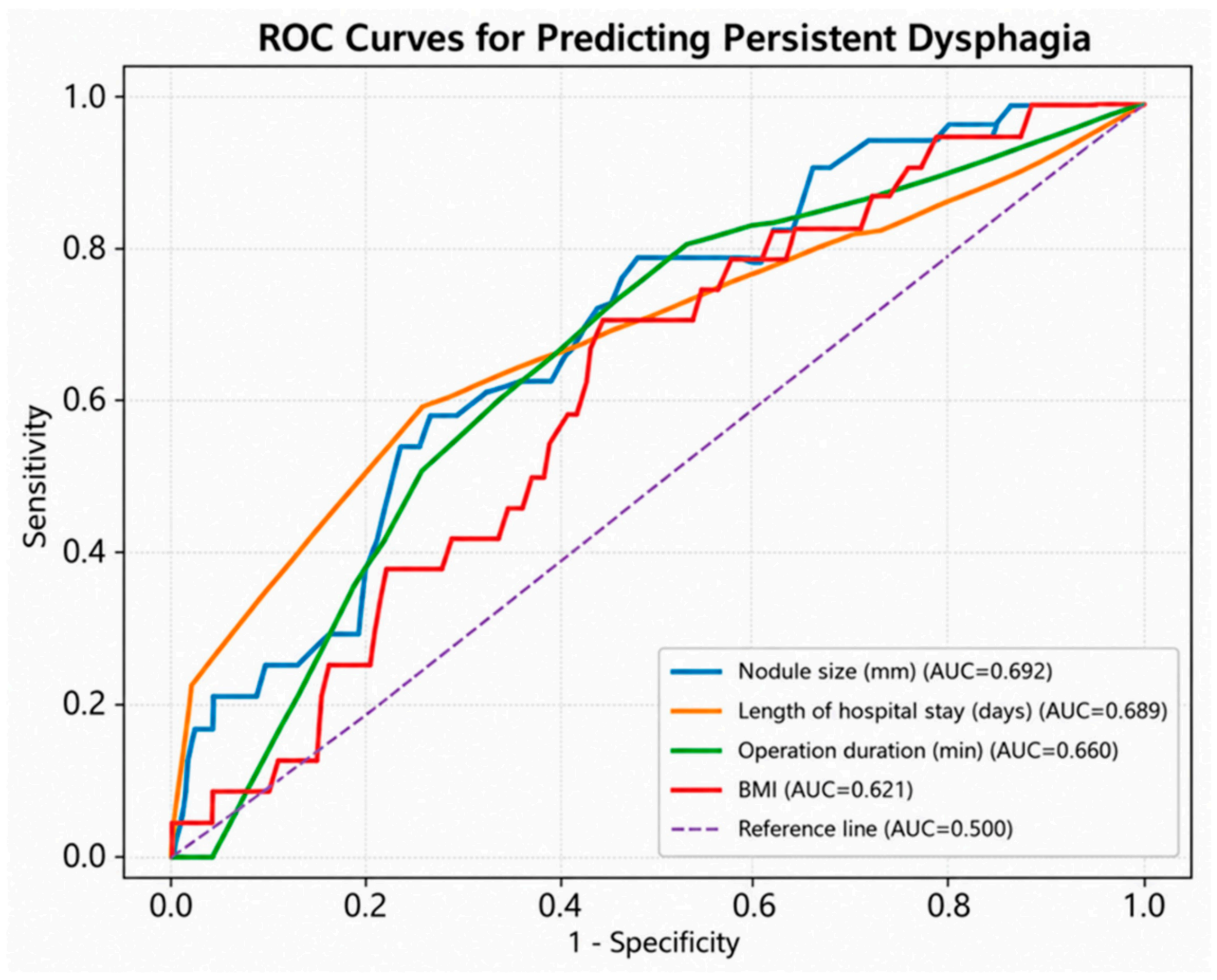
ROC curves demonstrating the ability of nodule size, surgery duration, length of hospital stay, and BMI to predict persistent patient-reported swallowing symptoms.

The ability of continuous variables to discriminate between cases of persistent patient-reported swallowing symptoms was evaluated using ROC analysis. Nodule size, length of hospital stay, and duration of surgery demonstrated significant discriminatory performance. The AUC value for BMI was at the borderline level of significance (see Table 5, Figure 2). To evaluate the stability of the multivariate predictive model in greater detail, internal validation was performed using the 5-fold cross-validation method. The model was demonstrated to maintain its discriminatory performance, with an average cross-validated AUC value of 0.801 and an apparent value of 0.833. These findings suggest that the model is sufficiently stable and that the risk of overfitting is limited.

## 4. Discussion

This study determined changes in postoperative swallowing function over time before and following thyroidectomy. It primarily demonstrated that dysphagia decreased significantly during the follow-up period. However, the presence of preoperative and postoperative dysphonia was found to be an independently associated factor for long-term persistent patient-reported swallowing symptoms. Additionally, parameters reflecting surgical complexity, such as nodule size, operative time, and length of hospital stay, were associated with persistent patient-reported swallowing symptoms.

This suggests that dysphagia following thyroidectomy can occur even in the absence of nerve damage [14,15]. Sinagra et al. reported that post-thyroidectomy dysphagia is a multifactorial process involving surgical manipulation, laryngotracheal mobilization, postoperative fibrosis, and changes in esophageal motility [16]. Similarly, another study demonstrated that swallowing problems can occur even in patients with normal laryngeal mobility [17]. Our current study also observed that the development of dysphagia cannot be explained solely by anatomical nerve damage, with laryngeal dysfunction playing a role.

In our study, the T-EAT-10 scores showed a decrease during the postoperative follow-up period. In particular, a substantial decline in dysphagia scores was evident at the 6- and 12-month postoperative points. This finding is consistent with previous studies that have reported that dysphagia symptoms improve over time in most patients following thyroidectomy [18,19]. Other studies have reported that voice and swallowing symptoms tend to be more pronounced in the early postoperative period but improve progressively during long-term follow-up [20,21]. In our study, dysphagia scores were also found to decrease, particularly from the 6-month mark onwards. When interpreting these findings, it is important to consider that dysphagia symptom outcomes are assessed using the T-EAT-10 questionnaire, reflecting patient-reported symptoms rather than a confirmed physiological impairment. Patient-reported symptoms are highly valuable but do not necessarily confirm a physiological swallowing impairment. It should be considered that in patients after thyroidectomy, frequent throat discomfort, globus changes, and voice alterations may affect patient responses.

Patients experienced compressive symptoms and swallowing difficulties even before surgery. The literature shows that multinodular goiter and large nodules are associated with preoperative dysphagia [22]. Furthermore, some studies have suggested that thyroid pathology itself may impair swallowing function by mechanically compressing the laryngopharyngeal region [23]. Therefore, dysphagia scores in the early postoperative period may be related to the balance between postoperative improvement and inflammatory changes following surgery. Although item-level analyses have not been performed, patients with dysphonia are expected to have a greater impact on the T-EAT-10 items that assess swallowing effort and the sensation of food lodged in the throat due to common laryngopharyngeal mechanisms.

In multivariate analyses, postoperative dysphonia was identified as the strongest independently associated factor for persistent swallowing symptoms. One possible explanation is the close anatomical and neurophysiological relationship between swallowing and phonation. However, the present study was not designed to investigate these mechanisms directly, and swallowing outcomes were assessed solely using the patient-reported questionnaire without objective dysphagia or dysphonia. Laryngeal coordination disorders, pharyngeal phase dysfunction, and inadequate glottic closure, which develop following involvement of the recurrent laryngeal nerve and the superior laryngeal nerve, can affect swallowing functions [24]. It was demonstrated by Lombardi et al. in long-term follow-up studies that postoperative voice changes are closely associated with dysphagia symptoms [1]. Postoperative dysphonia may reflect underlying laryngeal dysfunction rather than being a direct cause of persistent patient-reported dysphagia symptoms. Therefore, the observed relationship would be better interpreted in the context of laryngopharyngeal mechanisms that play a common role in voice and swallowing functions.

Our study also demonstrated that preoperative dysphonia is an independent risk factor. This suggests that laryngeal or functional abnormalities present prior to surgery may affect postoperative recovery. Nam et al. reported that laryngeal evaluations prior to thyroidectomy are important and that silent vocal cord dysfunction may go unnoticed [25,26]. For this reason, preoperative functional assessments are particularly important for symptomatic patients.

ROC analyses revealed threshold values of 29 mm for nodule size and 100 min for operation time. Larger nodule size and prolonged operation time may reflect increased surgical complexity and more intensive tissue manipulation. Hillenbrand et al. reported that more extensive surgical procedures may increase the risk of postoperative swallowing dysfunction (2). Similarly, previous systematic reviews have emphasized that the extent of surgery and tissue manipulation play a role in the development of dysphagia (3, 5). However, the discriminatory performance of these parameters remains limited, with AUC values below 0.70 indicating that they are not strong predictive markers in isolation. Therefore, these threshold values should be interpreted with caution and are considered to be more meaningful when employed alongside other clinical risk factors rather than evaluated as an independent associated factor. In addition, internal cross-validation analysis has demonstrated that the model’s performance remains distinctive. This finding supports the reliability and robustness of the multivariate model, despite the relatively limited number of patients who develop persistent swallowing symptoms. The identified exploratory threshold values, when interpreted together with other clinical risk factors, may serve as approximate clinical markers to support risk stratification rather than as strong predictive thresholds. Additionally, although internal cross-validation demonstrated acceptable model stability, the relatively small number of patients with persistent swallowing symptoms may have increased the risk of overfitting.

This study found that the presence of malignant pathology was independently associated with higher dysphagia scores in univariate analyses and that it remained an independent associated factor of persistent swallowing symptoms in multivariate analyses. However, this association should not be attributed directly to the malignancy itself. Patients with malignant thyroid disease may undergo more extensive surgical dissection, increased tissue manipulation, central neck dissection, and technically more complex surgical procedures. All of these factors can adversely affect postoperative swallowing function. Cases involving dense fibrotic planes, extensive vascularity, retrosternal extension, tissue adhesions, or disruption to anatomical dissection planes were classified as difficult dissections. However, difficult dissection, together with other unmeasured confounding factors such as laryngopharyngeal reflux and psychological factors, may have influenced persistent patient-reported dysphagia symptoms.

Previous studies have shown that the extent of surgery and manipulation of perilaryngeal tissues can contribute to postoperative functional impairment [5]. Therefore, the relationship between malignancy and persistent patient-reported dysphagia symptoms must be interpreted with caution, as malignancy may not play a role as an independent biological risk factor, but rather as an indicator of increased surgical complexity.

In essence, while dysphagia following thyroidectomy usually resolves over time, the presence of preoperative and postoperative dysphonia appears to be a strong indicator of persistent patient-reported swallowing symptoms. Furthermore, parameters reflecting surgical complexity, such as nodule size and operative time, may increase the risk of dysphagia. Therefore, long-term evaluation of swallowing function and preservation of laryngeal function are essential in patients undergoing thyroidectomy.

### Limitations

This study has some limitations. Dysphagia outcomes were measured using the T-EAT-10 scale, which assesses patient-reported symptoms. Therefore, the primary outcome reflects patients’ perceived symptoms rather than objectively confirmed dysphagia. Furthermore, postoperative throat discomfort, globus sensation, and voice changes following thyroid surgery may have influenced responses to the questionnaire. Although dysphonia and dysphagia were assessed as separate variables in the present study, both reflect aspects of laryngopharyngeal function. Therefore, some T-EAT-10 items may partially capture overlapping laryngopharyngeal symptomatology rather than dysphagia alone. The clinical risk factors identified in our study, while not definitive predictors, may aid in risk stratification for persistent patient-reported swallowing symptoms after thyroid surgery.

Furthermore, potentially confounding factors such as laryngopharyngeal reflux, psychological factors, and other conditions that may influence swallowing symptoms were not included in the analyses, representing another limitation of this study. Although multivariate analysis was performed, the relatively small number of patients with persistent patient-reported swallowing symptoms may have limited the implications.

Another limitation of this study was that postoperative swallowing symptoms were assessed at the scheduled follow-up visits whenever possible. For patients who were unable to attend follow-up, outcomes were obtained retrospectively through patient recall. Furthermore, the inclusion of all patients within the defined range due to the retrospective design may have increased the risk of both type I and type II statistical errors and limited the stability of the regression coefficients.

Future prospective multicenter studies combining patient-reported outcome measures with objective instrumental swallowing assessments and standardized voice evaluations, while also incorporating standardized measures of surgical complexity and other potential patient-related confounding factors, are warranted to validate these findings.

## 5. Conclusions

These findings emphasize the importance of evaluating long-term swallowing function following thyroidectomy. Surgical strategies aimed at preserving laryngeal function and close postoperative follow-up may therefore help to reduce the risk of persistent patient-reported swallowing symptoms. Although most patients experience a reduction in symptoms of dysphagia following thyroidectomy, some patients may develop persistent long-term dysphagia. This study demonstrated that preoperative and postoperative dysphonia are particularly associated with persistent patient-reported swallowing symptoms. Additionally, parameters reflecting surgical complexity, such as nodule size, operative time, and length of hospital stay, were found to be associated with the development of dysphagia. The clinical exploratory threshold values identified in ROC analyses may be useful in predicting which patients are at high risk. Given their modest discriminatory performance, these thresholds should be interpreted as supportive markers.

## Figures and Tables

**Table 1:** Demographic and clinical characteristics of patients.

Patient Characteristics	Mean ± SD/*n* (%)
Demographic	
Age (years)	52.67 ± 11.14 Median (range): 53 (27–78)
BMI (kg/m^2^)	25.99 ± 5.48
Female, *n* (%)	219 (78.2)
Male, *n* (%)	61 (21.8)
Smoking, *n* (%)	70 (25.0)
Clinical	*n* (%)
Presence of comorbidities	146 (52.1)
Benign pathology	202 (72.1)
Malignant pathology	78 (27.9)
Papillary thyroid microcarcinoma	40 (14.3)
Papillary thyroid carcinoma	26 (9.3)
Nodule size < 20 mm, *n* (%)	72 (25.7)
Nodule size 20–40 mm, *n* (%)	144 (51.4)
Nodule size > 40 mm, *n* (%)	64 (22.9)
Total	280

Abbreviations: SD, standard deviation; BMI, body mass index; mm, millimeter; *n*, number; %, percent.

**Table 2:** T-EAT-10 scores over time before and following thyroidectomy (*n:* 280).

Time Point	Mean ± SD (Min–Max)	Min–Max
Preoperative	1.86 ± 3.04	0–12
1st month postoperative	1.98 ± 2.34	0–12
6th month postoperative	0.72 ± 1.53	0–12
12th month postoperative ^1^	0.45 ± 1.19	0–7

^1^ Twelve months were available for 279 patients. Abbreviations: T-EAT-10, Turkish Eating Assessment Tool-10; SD, standard deviation.

**Table 3:** Comparison of clinical factors related to persistent patient-reported swallowing symptoms.

Variable	No Dysphagia (*n* = 255) Mean ± SD	Dysphagia Present (*n* = 24) Mean ± SD	*p*-Value
Thyroid weight (g)	86.43 ± 79.81	76.69 ± 59.40	0.737
Length of hospital stay (days)	1.28 ± 0.50	1.83 ± 0.82	<0.001
Operation duration (minute/dk)	97.96 ± 9.08	102.92 ± 8.06	0.005
TSH	2.09 ± 4.13	1.76 ± 1.41	0.513
Nodule size (mm)	28.95 ± 14.85	40.38 ± 17.35	0.002
BMI	25.83 ± 5.47	28.05 ± 5.10	0.050
Age	52.69 ± 11.11	52.25 ± 11.82	0.934

Abbreviations: SD, standard deviation; BMI, body mass index; mm, millimeter.

**Table 4:** Multivariate logistic regression analysis for persistent patient-reported swallowing symptoms.

Variable	OR	%95 CI	*p*-Value
Preoperative dysphonia	4.40	1.42–13.66	<0.010
Postoperative dysphonia	17.54	4.13–74.56	<0.001
BMI	1.10	1.01–1.21	0.030
Malignant pathology	3.36	1.22–9.26	0.019
Nodule size (mm)	1.03	0.99–1.06	0.106

Note: Model statistics: χ^2^ = 43.06, *p* < 0.001; Hosmer–Lemeshow χ^2^ = 11.19, df = 8, *p* = 0.191; Nagelkerke R^2^ = 0.322. Abbreviations: OR, odds ratio; CI, confidence interval.

**Table 5:** ROC analysis results for persistent patient-reported swallowing symptoms.

Variable	AUC (%95 GA)	Exploratory Threshold	Sensitivity (%)	Specificity (%)	*p*
Nodule size (mm)	0.692 (0.579–0.793)	29	79.2	52.5	0.002
Length of hospital stay (days)	0.689 (0.574–0.803)	2	58.3	74.5	0.002
Operation duration (min)	0.660 (0.554–0.756)	100	79.2	49.4	0.011
BMI	0.621 (0.514–0.724)	26.6	70.8	56.5	0.056

Abbreviations: AUC, area under the receiver operating characteristic (ROC) curve; BMI, body mass index. Note: Optimal exploratory thresholds were determined using the Youden index.

## Data Availability

The datasets generated and/or analyzed during the current study are not publicly available due to privacy but are available from the corresponding author upon reasonable request.

## Abbreviations

The following abbreviations are used in this manuscript:

SD	Standard Deviation
BMI	Body Mass Index
T-EAT-10	Turkish Eating Assessment Tool-10
AUC	Area Under the Curve
CI	Confidence Interval
